# An open participatory protocol for the ecological development and validation of the interactional disruption profile

**DOI:** 10.3389/fpsyg.2026.1889253

**Published:** 2026-10-05

**Authors:** Guillaume Broc, Victor Vattier, Richard C. Schmidt, Delphine Capdevielle, Ludovic Marin, Stéphane Raffard

**Affiliations:** 1 Psychology Department, University of Montpellier Paul Valery, Montpellier, France; 2 ICARES French Institute for Comprehension, Anticipation and Intervention Research in Environment and Global Health, University of Montpellier Paul Valery, Montpellier, France; 3 EuroMov Digital Health in Motion, Montpellier, France; 4 College of the Holy Cross, Worcester, MA, United States; 5 University Department of Adult Psychiatry, CHU Montpellier, Montpellier, France

**Keywords:** clinical assessment, context-dependent constructs, ecological validity, interactional disruption, nonverbal communication, open participatory protocol, psychological testing

## Abstract

This article presents an open participatory protocol for the ecological development and validation of the Interactional Disruption Profile, a clinical assessment instrument intended to assess observable nonverbal behaviors that may contribute to interactional disruption during real-time interactions. Grounded in current standards for psychological testing, the protocol integrates psychometric, ecological, and implementation-related considerations throughout instrument development. The procedure is structured into three phases: (1) test development (project governance, intended use, construct definition, administration specifications, and candidate item generation); (2) test validation (content validation, pretesting, psychometric evaluation); and (3) test production (manual drafting & writing of the test manual). A Steering Committee involving researchers, clinicians, patients, and relatives will supervise the iterative co-construction of the instrument and contribute to methodological decision-making throughout development. Particular emphasis is placed on ecological validity and on explicitly documenting the methodological trade-offs involved in balancing psychometric robustness, contextual sensitivity, usability, and implementation constraints. Rather than prescribing the future characteristics of the instrument, the article proposes an open participatory protocol that structures the iterative development and validation process by ensuring that decisions are progressively informed by evidence, deliberated, documented, and refined. Beyond providing an open protocol suited to capturing context-dependent constructs such as complex interactional phenomena, the article illustrates how psychological testing standards may be applied within an ecologically grounded development and validation process.

## 1 Introduction

### 1.1 Nonverbal disruption in social interaction

Nonverbal communication plays a central role in social functioning through facial expressions, gestures, gaze, posture, interpersonal distance, and vocal prosody (Burgoon et al., 1989; Hall, 2001; Mandal, 2014). These processes regulate interpersonal coordination and interactional coherence. Consequently, disturbances in nonverbal communication may impair empathy, mutual understanding, social coordination, and interactional fluidity (Brüne et al., 2008). Research has documented nonverbal disturbances across several clinical conditions, including schizophrenia, autism spectrum disorder, and social anxiety disorder (Del-Monte et al., 2013; Georgescu et al., 2020; Lavelle et al., 2014; Walther et al., 2015). These manifestations may include reduced expressivity, atypical gaze, altered synchronization, diminished responsiveness, repetitive gestures, unusual postures, or poorly timed behaviors. Interactional difficulties may result from reduced or absent signals, but also from behaviors that are excessive, intrusive, or incongruent with the context (Hardin, 1980; Mittal and Walker, 2007; Walker et al., 1999).

Whether a behavior is experienced as disruptive depends on its timing, intensity, cultural meaning, and on the expectations and responses of the interaction partners (Timmermans and Schilbach, 2014). In this article, interactional disruption is therefore provisionally defined as a context-dependent difficulty in reciprocity, mutual understanding, comfort, or engagement to which observable behavior may contribute. The focus is on what occurs during a specific interaction and on its perceived impact on the partner’s experience. This impact remains partly subjective, as the same behavior may be experienced differently across individuals and situations. Interactional disruption is not treated as an inherent property of a person or behavior, and no assumptions are made about diagnosis, medication, stress, intention, personality, or other possible causes.

Most existing approaches examine nonverbal behaviors separately, although disruption may emerge from the way several signals combine, unfold, and adjust during real-time exchanges (Atzil-Slonim and Tschacher, 2020; Pan and Cheng, 2020). This raises a central methodological question: how can observable behavior be analyzed systematically without losing the subjective and context-dependent nature of the interactional disruption?

### 1.2 Limits of current assessment procedures

Several methods have been developed to assess nonverbal alterations and related psychopathological manifestations. Clinical scales such as the Positive and Negative Syndrome Scale (PANSS; Kay et al., 1987), the Clinical Assessment Interview for Negative Symptoms (CAINS; Kring et al., 2013), and the Scale for the Assessment of Negative Symptoms (SANS; Andreasen, 1989) mainly conceptualize nonverbal disruption through symptom severity, particularly reduced expressivity and social withdrawal. Other instruments focus on more specific behavioral dimensions, while ethological and technological approaches quantify gestures, facial activity, synchronization, and movement dynamics (Ekman and Friesen, 1978; Kupper et al., 2010; Lavelle et al., 2014; Riehle et al., 2017; Troisi, 1999). Despite their strengths, these approaches present important limitations for ecological clinical assessment. Clinical instruments often prioritize psychopathological categories over dynamic interactional processes, whereas observational and technological procedures may require resources incompatible with routine practice (Burns and Patrick, 2007). The methodological gap addressed in this article is therefore not the absence of instruments assessing nonverbal or interactional manifestations. Rather, it concerns how observable behaviors, their context of occurrence, and their possible interactional impact can be incorporated into an assessment procedure that remains psychometrically defensible, clinically interpretable, and feasible under intended conditions of use. Similar tensions arise whenever clinical instruments target phenomena that are context-dependent, dynamic, or difficult to separate from the situations in which they are expressed.

### 1.3 Ecological challenges in psychological testing

These limitations raise broader questions about ecological validity in psychological measurement, defined here as the extent to which assessment conditions, observed manifestations, and resulting interpretations adequately represent the target construct within its intended real-world contexts. For interactional disruption, this means assessing behavior in the interpersonal situations in which it acquires meaning, rather than assuming that observations made in a single clinical encounter necessarily represent difficulties arising in family, occupational, or other everyday interactions.

Ecological validity should nevertheless be distinguished from several related considerations. Feasibility concerns whether an assessment can be applied under its intended conditions, implementation concerns whether it can be adopted and maintained in practice, and consequences of testing concern the intended and unintended effects of its use. These dimensions may influence one another, but they raise distinct methodological questions.

Reports of instrument development often emphasize statistical indicators such as internal consistency, factor loadings, and model-fit indices more explicitly than assessment context, response processes, usability, or consequences of use (Gan et al., 2012; Truijens et al., 2019; Wong, 2006). Yet an instrument’s validity depends not only on statistical performance, but also on the conditions under which it is administered, interpreted, and used (Leach and Oakland, 2007; Uher, 2022). Even when satisfactory psychometric evidence is reported, guidance concerning administration procedures, acceptable contextual adaptations, required user competencies and interpretative boundaries may remain limited (Morgado et al., 2017). This may encourage users to treat validity as a stable property of the instrument rather than as a property of specific interpretations and uses. It may also lead clinicians to avoid the instrument, adapt its procedures informally, or interpret its results beyond their intended scope (Jensen-Doss and Hawley, 2010).

Users’ training and competencies may also influence how an instrument is administered and interpreted, particularly when ratings require familiarity with the construct and access to relevant contextual information (Uher, 2022). Ecological validity also depends on whether the assessment is conducted in situations where the target phenomenon is likely to occur. Likewise, development samples may not reflect the diversity of the populations and settings in which the instrument is intended to be used, limiting confidence in the generalizability of its psychometric properties (Green et al., 2011).

International guidelines such as the *Standards for Educational and Psychological Testing* (American Educational Research Association et al., 2014), the *Guidelines for International Testing* (International Test Commission, 2018), and the *ITC Guidelines for Test Use* (Bartram and Hambleton, 2016; International Test Commission, 2001) emphasize that validity evidence should extend beyond test content, internal structure, or relations to other variables. It should also include qualitative and contextual dimensions such as response processes and consequences of testing, especially regarding the soundness of score interpretations for responsible in situ use (see Table 1). Within this perspective, stakeholder participation, particularly from clinicians and patients, is encouraged throughout instrument development and validation.

**Table 1:** Main sources of validity evidence according to the standards for educational and psychological testing and their ecological implications for test development and use.

Validity evidence	Main question addressed	Implications
(1) Evidence Based on Test Content	Do the items, instructions, and response formats adequately represent the construct and its relevant contexts of expression?	*If supported*, the instrument captures the core dimensions and contextual manifestations of the construct. *If weak or unsupported*, important behaviors, situations, or contextual nuances may be omitted or poorly represented.
(2) Evidence Based on Response Processes	Do respondents and users understand, interpret, and engage with the test materials and administration procedures as intended?	*If supported*, scores are more likely to reflect the targeted construct as experienced or observed in practice. *If weak or unsupported*, scores may reflect misunderstanding, cognitive burden, literacy issues, or inappropriate administration conditions.
(3) Evidence Based on Internal Structure	Does the empirical structure of the test correspond to the theoretical and clinical organization of the construct?	*If supported*, the scale provides coherent and interpretable scores. *If weak or unsupported*, dimensions may be statistically unstable, clinically unclear, or disconnected from real-world manifestations of the construct.
(4) Evidence Based on Relations to Other Variables	Do scores relate to other constructs, behaviors, or outcomes in theoretically and clinically expected ways?	*If supported*, the instrument is meaningfully situated within its nomological and clinical network. *If weak or unsupported*, the scale may lack specificity, overlap excessively with neighboring constructs, or fail to capture clinically relevant functioning.
(5) Evidence Based on Consequences of Testing	Does the instrument support appropriate, responsible, and context-sensitive use in practice?	*If supported*, the instrument can guide interpretation and use within explicit boundaries. *If weak or unsupported*, risks of misuse, overpathologization, inappropriate adaptation, or harmful clinical decisions may increase.

Translating these principles into psychometric development remains difficult when the target construct is interactional, context-dependent, and only partially stabilized conceptually. Such constructs require developers to consider not only psychometric performance, but also the contexts in which the phenomenon is expressed, the conditions under which it is assessed, the perspectives involved in its interpretation, and the consequences of its use. An adaptable and explicitly documented methodological pathway is therefore needed to organize these decisions without treating contextual variability as a secondary concern.

### 1.4 Objective

The primary objective of this article is to propose an open participatory protocol for the ecological development and validation of the Interactional Disruption Profile (IDP), a clinical assessment instrument intended to assess socially disruptive nonverbal behaviors during real-time interactions. More specifically, the article aims to show how psychological testing standards may be applied within an ecologically grounded development and validation process, particularly for the assessment of context-dependent constructs such as complex interactional phenomena.

## 2 Epistemological positioning

### 2.1 Ecological validity as a foundational psychometric issue

Ecological validity is often treated as a secondary property examined after psychometric validation. However, in the context of interactional disruption, it may instead constitute a foundational issue affecting all stages of instrument development (Beckman et al., 2005; American Educational Research Association et al., 2014). Decisions concerning item wording, observational context, administration conditions, scoring procedures, or interpretative recommendations may alter the meaning and boundaries of the construct itself (Leach and Oakland, 2007; Uher, 2022). Interactional behaviors are particularly sensitive to contextual variability because their meaning depends on timing, reciprocity, interpersonal expectations, and situational norms (Timmermans and Schilbach, 2014). A gesture or facial expression may contribute to interactional difficulty in one context while remaining acceptable in another. Psychometric standardization alone may thus be insufficient to capture the ecological complexity of such behaviors (Truijens et al., 2019).

### 2.2 Negotiating methodological trade-offs

Developing ecologically grounded clinical instruments involves continuous decisional tensions between partially competing objectives. Increasing the number of items may improve internal consistency while reducing usability and increasing cognitive burden. Highly standardized procedures may strengthen reliability but reduce the flexibility needed in real-world interactions. Conversely, excessive flexibility may compromise score comparability and interpretative coherence (Van de Vijver, 2016). Similarly, maximizing ecological realism may complicate factorial stability, whereas stricter psychometric optimization may distance the instrument from the phenomena it intends to assess. Psychometric development should therefore not be conceived as a linear accumulation of validity indicators, but as a process of negotiated methodological trade-offs requiring explicit documentation and justification throughout development (American Educational Research Association et al., 2014).

### 2.3 Participatory and iterative development

Iterative and participatory approaches combining top-down procedures (literature reviews and theoretical analyses) with bottom-up procedures (clinical observations, stakeholder consultation, cognitive interviews, and participatory decision-making) are increasingly recommended to improve the contextual relevance, feasibility, and responsible deployment of clinical instruments (Duea et al., 2022; Skivington et al., 2021; Vaughn and Jacquez, 2020). From this perspective, instrument development would benefit from the supervision of a Multidisciplinary Steering Committee (e.g., composed of researchers, clinicians, and patient representatives). Such a reflexive approach promotes a more comprehensive understanding of the instrument by documenting its developmental history, underlying rationale, and intended functions, thereby supporting its informed deployment and context-sensitive adaptation.

## 3 Materials and procedural resources for IDP development and validation

The following section presents the proposed open participatory protocol for the ecological development and validation of the Interactional Disruption Profile (IDP). Figure 1 first presents an integrative metrological framework synthesizing the epistemological principles developed above with current psychometric standards (American Educational Research Association et al., 2014) and existing methodological guidance for instrument development (e.g., Boateng et al., 2018; Irwing and Hughes, 2018; Kyriazos and Stalikas, 2018). Rather than proposing a new psychometric framework, it provides an organizing representation of the methodological principles underpinning the protocol. These principles are then operationalized through the successive development, validation, and production phases described in Tables 2–4.

**Figure 1:**
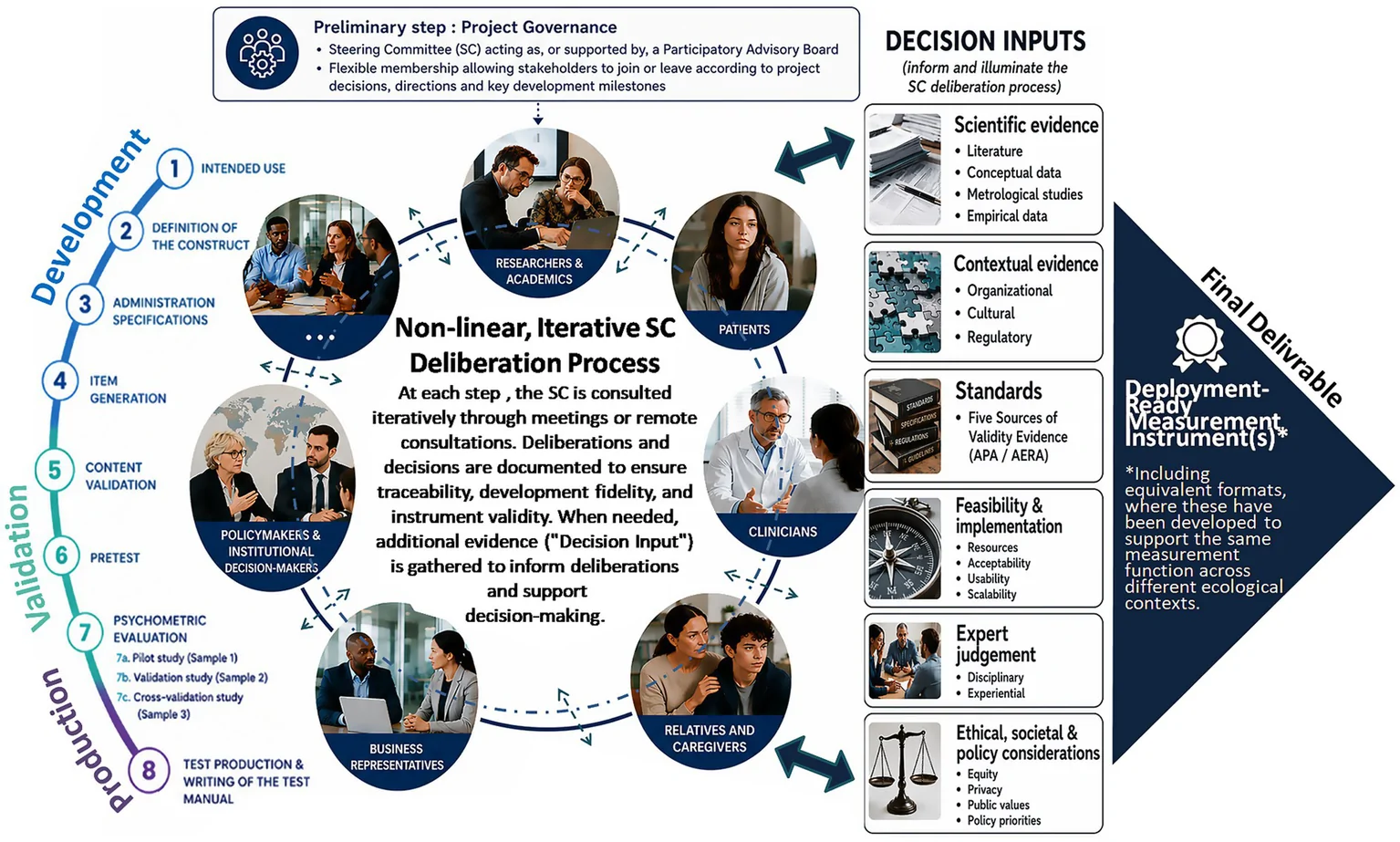
General framework for the ecologically based development and validation of measurement instruments.

**Table 2:** Open participatory protocol for the ecologically-based development and validation of the IDP instrument (development phase).

Development phase				
Open protocol step	SC non-linear deliberation process (key questions)	Decision inputs (illustrative methods and evidence sources)	Illustrative IDP development scenarios	Validity-informed decision criteria
Preliminary step: Project Governance *Objective:* Establish a participatory governance structure ensuring stakeholder representation, transparent deliberation, methodological oversight, and decision traceability throughout instrument development.	Which stakeholders and expertise should be represented throughout development (patients, relatives, clinicians, researchers, health policymakers, healthcare managers.)? How should membership evolve according to project needs? How should disagreements be managed and documented? When should additional evidence (“Decision Inputs”) be requested before reaching a decision? Which decisions require collective consensus versus delegated expertise? How is decision traceability ensured?	Stakeholder mapping Governance framework Partnership charter (shared values and commitments) Facilitation methods Participatory research guidance	Governance including at least researchers specializing in social interactions, clinicians with expertise in psychopathology and social disorders, psychometricians, implementation scientists, and patient and family representatives. Flexible SC membership (e.g., graphic or app designers for production deliverables).	TC: Representative governance supports comprehensive construct coverage across relevant populations, contexts, and stakeholder perspectives. CoT: Representative governance is needed to foresee risks and unintended consequences while limiting institutional, political, or stakeholder influence that could unduly constrain the instrument’s purpose, scope, adaptability, or use.
1. Intended Use *Objective:* Define measurement functions and intended clinical/research use; discuss the consequences of appropriate and inappropriate use for patients and their ecosystem.	Which measurement function(s)? (screening, assessment, monitoring, research.) Which target users (clinicians, researchers, caregivers.) and populations? Which contexts of use? (hospital, rehabilitation, home, personal, social, professional.) Which stakeholder needs should be prioritized? Which are mutually beneficial, merely compatible (commensal), or potentially conflicting (dysbiotic)? Which trade-offs between feasibility, precision and utility? Which inappropriate uses should be anticipated? Which consequences for patients and their ecosystem?	Environmental scan Existing instrument review (gaps and unmet needs) Stakeholder interviews and focus groups (expected uses and needs) RAND/UCLA Appropriateness Method (appropriateness of intended-use scenarios)	Routine assessment of social interactions during clinical follow-up. Context-specific versions (healthcare; personal, social, professional), if justified. Single instrument supporting clinical assessment, intervention planning, crisis prevention, self-management, longitudinal progress monitoring, and research.	TC: Intended uses, target users, populations, contexts, and measurement functions are sufficiently specified to guide subsequent construct definition, administration, and item development. CoT: Intended uses and interpretive boundaries are sufficiently delimited to prevent inappropriate interpretation, decision-making, or use beyond the instrument’s intended scope.
2. Construct Definition *Objective:* Define the construct, its conceptual boundaries, dimensions, and nomological network.	Which phenomena belong to the construct and which should be excluded? Which level(s) should be measured (e.g., behaviors, interactional processes, outcomes)? Which conceptual organization best represents the construct (e.g., unidimensional, multidimensional, hierarchical, profile-based)? Which characteristics should remain invariant, and which may legitimately vary across time, populations, cultures, or contexts? Which conceptual trade-offs best balance comprehensiveness, parsimony, interpretability, flexibility, and intended use?	Scoping/systematic literature review Existing instrument review (construct coverage and limitations) Concept analysis/concept mapping Stakeholder interviews and focus groups (construct representation) Cross-cultural evidence	Interactional disruption could be retained as the target construct, independently of its underlying causes or clinical diagnoses. Reduced and excessive/incongruent manifestations could be retained as provisional descriptive categories. A total score, subscales, or an interactional profile could be retained to represent the construct.	TC/RoV: The construct boundaries, dimensions, nomological network, and contextual manifestations form a sufficiently explicit and operationalizable model to support subsequent item generation (Step 4) and hypothesis-driven examination of relations with external variables (Step 7b), while preventing construct contamination or overlap with adjacent constructs. CoT: The construct is sufficiently delimited to prevent overpathologization or interpretation beyond its intended conceptual scope, consistently with the uses and safeguards defined in Step 1. RP: The construct definition is sufficiently parsimonious and operationally stable to be translated into feasible administration specifications and candidate items (Steps 3 and 4) without unnecessary complexity or respondent and user burden.
Step 3. Administration Specifications *Objective:* Specify how the measurement functions will be operationalized; Determine who will provide information, how and under which conditions.	Who should provide information (e.g., self-report, proxy, clinician, peer, multiple informants)? Which administration mode(s) best support the intended use (e.g., questionnaire, interview, observation, simulated or elicited situations)? Which administration conditions should be standardized, adapted, or tailored across settings, populations, cultures, or contexts? Which operational trade-offs best balance flexibility, standardization, feasibility, accessibility, and measurement comparability?	Review of existing administration methods and modalities Implementation, feasibility and acceptability investigation Analysis of contextual and logistical constraints Experience-based co-design workshops Ideation methods (tailored, dynamic, context-adaptive administration formats) Regulatory and professional recommendations	Self-report, proxy-report, clinician-report, and/or multi-informant approaches could be combined within a modular assessment strategy. Hybrid administration (e.g., structured interview combined with observation, such as CAINS) could be considered depending on the intended use and context Clinical interactions could be video-recorded to support standardized longitudinal capture and objective documentation of IDP changes across repeated assessments. Context-adaptive (e.g., modular) administration procedures could be retained while preserving equivalent measurement functions across administration modalities. Different administration modalities (e.g., paper-and-pencil, clinician-rated, smartphone-based) and inclusive formats (e.g., image-based formats for lower literacy populations) could be considered	TC: Alternative administration modalities preserve equivalent measurement functions (see Steps 1 & 2) to improve further intended score interpretation and comparability across respondents and contexts RP/CoT: Administration choices minimize foreseeable barriers, inequities, inappropriate use, and respondent burden while preserving accessibility and intended interpretation consistently with the intended uses and safeguards established in Step 1
Step 4. Candidate Item Generation *Objective:* Generate candidate items representing the construct and its contextual manifestations.	Which observable indicators best represent each dimension of the construct?• Which item generation strategies should be combined (e.g., deductive, inductive, participatory)? How should the administration strategy defined in Step 3 be operationalized into item formats, response options, coding procedures, and scoring principles? Which wording best captures observable phenomena while avoiding causal, diagnostic, or stigmatizing interpretations? Which contextual information should accompany item administration or interpretation? Which conceptual and operational trade-offs best balance comprehensiveness, clarity, redundancy, ecological validity, respondent burden, and clinically meaningful discrimination?	Literature review and extraction of candidate indicators Qualitative methods (e.g., interviews, observations, focus groups) Existing instruments and item mapping Participatory co-design and ideation workshops Item writing guidelines and best practice recommendations	Candidate items could document observable behaviors of both interaction partners, their reciprocal responses, and their contribution to interactional disruption in context (e.g., MISC-inspired interaction coding). Alternative coding and scoring systems (e.g., ordinal, weighted, nonlinear) could be compared to better reflect the clinical significance of disruption severity. Video-recorded interactions could be processed and segmented into standardized observation units to systematically characterize behavioral responses together with their interactional context and consequences, thereby supporting candidate item generation. Free-text observation fields could contextualize structured ratings to support clinically meaningful interpretation.	TC: The proposed operationalization remains consistent with the construct (Step 2) and the administration strategy (Step 3). RP: Item wording, coding procedures, and scoring rules are expected to function as intended under the administration conditions defined in Step 3. CoT: Item wording, coding procedures, scoring rules, and interpretation remain consistent with the intended consequences and safeguards defined in Steps 1 and 3.

SC = Steering Committee; TC = Evidence Based on Test Content; RP = Evidence Based on Response Processes; IS = Evidence Based on Internal Structure; RoV = Evidence Based on Relations to Other Variables; CoT = Evidence Based on Consequences of Testing.

**Table 3:** Open participatory protocol for the ecologically-based development and validation of the IDP instrument (validation phase).

Validation phase					
Open protocol step	SC non-linear deliberation process (key questions)		Decision inputs (illustrative methods and evidence sources)	Illustrative IDP development scenarios	Validity-informed decision criteria
Step 5. Content Validation *Objective:* Verify that the instrument adequately represents the construct in its relevant contexts.	Does the current instrument adequately represent the construct, its dimensions, and contextual manifestations? Should dimensions, domains, hierarchical organization, or candidate content be added, revised, merged, or removed? Which conceptual and operational trade-offs best balance construct coverage, clarity, redundancy, feasibility, and instrument length? Which wording or content could lead to ambiguity, misunderstanding, construct contamination, or stigmatization?		Expert consensus methods (e.g., TRIAGE, Delphi, Nominal Group Technique) RAND/UCLA Appropriateness Method (content validity against intended-use requirements) Content Validity Ratio (CVR) or Content Validity Index (CVI) Qualitative expert feedback and consensus discussions Face validity assessment with intended users and stakeholders Item sorting/Item-to-construct matching	At least one TRIAGE consensus method involving 6–10 experts external to the project and specialized in social interactions and social disruption will be conducted. The IDP structure could then be revised by adding, removing, merging, or splitting dimensions, domains, or items. Hierarchical organization, wording, or content could be refined to improve representativeness, comprehensiveness, and clarity.	TC: The soundness of the content-related decisions established during Steps 1–4 is now put to the test by evaluating the instrument’s content validity. RP, IS, RoV, CoT: External expert feedback may additionally inform these other sources of validity by identifying issues requiring subsequent empirical evaluation or further deliberation.
Step 6. Pretest *Objective:* Verify the clarity of instructions, items, and response format; assess acceptability, usability or even implementation through user feedback.	Do intended users understand and apply the instrument as intended? Which practical constraints or usability issues require adaptation of the instrument? Which wording, format, scoring, or administration procedures should be modified? Which revisions are necessary before psychometric validation?		Cognitive interviews (e.g., think-aloud, verbal probing) Observation of instrument administration User-centered instrument refinement methods (e.g., task analysis, contextual inquiry, iterative prototyping) Usability testing Qualitative analysis of response processes	As the instrument would most likely be hetero-administered, cognitive interviews could be conducted with approximately 6–10 clinicians and/or relatives, either during instrument administration or during debriefing sessions based on video-recorded clinical interviews. Instructions, items, response format, scoring procedures, or administration procedures could subsequently be revised and, if necessary, re-evaluated through additional cognitive interviews.	RP: The soundness of the response process-related decisions established during Steps 3–4 is now put to the test by evaluating whether intended users can appropriately administer, score, and interpret the instrument as intended. TC, IS, RoV, CoT: Any modifications resulting from response process evaluation should be examined in light of these other sources of validity evidence to ensure that improving response processes does not inadvertently compromise content, internal structure, relations with other variables, or intended consequences.
Step 7. Psychometric evaluation	7a. Pilot study *Objective:* Preliminary evaluation of psychometric functioning and identification of issues requiring instrument refinement.	Does the instrument demonstrate acceptable preliminary psychometric functioning (sensibility, reliability and validity)? Which psychometric findings require refinement before validation? Which measurement/scoring model should be retained? How should earlier development decisions be reconsidered? How should contextual information be integrated into score computation and interpretation while preserving the intended measurement functions?	Core: Sensibility tests (e.g., Ferguson’s delta, Finlay’s D-index); reliability tests (e.g., test–retest reliability, inter-rater reliability, α, ω and AVE coefficients for internal consistency); indicator/component analyses; exploratory psychometric analyses appropriate to the proposed measurement model (e.g., EFA, IRT/Rasch models, G-Theory, where applicable). Additional (where appropriate): Alternative measurement/scoring model comparisons; responsiveness analyses; other psychometric evidence relevant to the intended use.	A first heterogeneous sample could compare alternative measurement/scoring architectures (e.g., latent dimensions, profile-based assessment or behavioral network analysis), identify clinically meaningful behavioral constellations, and guide instrument refinement. Three to four raters could independently assess approximately 40 contextualized behavioral video segments to standardize the assessment procedure, evaluate inter-rater reliability, and compare latent versus profile-based scoring approaches. When several administration formats are retained (e.g., observational, proxy-report or multi-informant), their measurement equivalence could be evaluated according to the intended measurement functions. The instrument could subsequently be refined.	IS: The retained measurement model is supported by appropriate psychometric evidence, while remaining conceptually coherent with the intended use (Step 1), construct definition (Step 2), and intended administration (Step 3). TC, RP, RoV & CoT: Any revisions prompted by preliminary psychometric findings should remain consistent with these other sources of validity evidence.
	Step 7b. Validation study *Objective:* Confirm the retained measurement model and evaluate hypothesized relations with external variables.	See Step 7a, plus: Are hypothesized relations with external variables supported?	See Step 7a, replacing exploratory with confirmatory or replication-oriented psychometric analyses appropriate to the retained measurement model (e.g., CFA, IRT/Rasch calibration, G-Theory, where applicable), and adding tests of hypothesized relations to other variables.	The retained measurement/scoring architecture (e.g., latent dimensions, behavioral profiles or network structure) could be evaluated in an independent sample. Hypothesized associations could be tested according to the nomological network established in Step 2.	IS: Confirmation or non-confirmation of the objectives established in Step 7a. RoV: Evidence based on relations to other variables is evaluated through tests of convergent, discriminant, criterion-related and/or predictive validity according to the nomological network established in Step 2. TC, RP & CoT: See Step 7a.
	Step 7c. Cross-validation study *Objective:* Evaluate the reproducibility of psychometric properties and establish score interpretation standards, where appropriate.	See Step 7b, plus: Are psychometric properties reproducible? How can score interpretation standards be established across different administration formats while preserving equivalent measurement functions?	See Step 7b, adding replication of sensibility and reliability estimates; equivalence testing across administration formats (e.g., ROC analyses, agreement/equivalence testing, where appropriate); reference/normative data (norming); cut-off, classification and/or meaningful-change analyses; and other evidence supporting score interpretation.	The retained measurement/scoring architecture and its associated score or profile interpretation could be independently replicated. Score interpretation standards (e.g., reference values, cut-offs or meaningful-change thresholds) could be established, where appropriate.	IS, RoV: Independent confirmation or non-confirmation of the objectives established in Steps 7a and/or 7b. TC, RP & CoT: See Step 7a.

SC = Steering Committee; TC = Evidence Based on Test Content; RP = Evidence Based on Response Processes; IS = Evidence Based on Internal Structure; RoV = Evidence Based on Relations to Other Variables; CoT = Evidence Based on Consequences of Testing.

**Table 4:** Open participatory protocol for the ecologically-based development and validation of the IDP instrument (production phase).

Production phase				
Open protocol step	SC non-linear deliberation process (key questions)	Decision inputs (illustrative methods and evidence sources)	Illustrative IDP development scenarios	Validity-informed decision criteria
Step 8. Test Production & Writing of the Test Manual (Implementation Planning) *Objective:* Packaging of the final instrument, including development of the test manual and implementation package/ensuring traceability of metrological decisions across the five sources of validity evidence to inform instrument interpretation, use, and implementation.	How should the accumulated validity evidence be triangulated to determine the instrument’s overall gradient of validity? Which version(s) of the instrument should be released? Which recommendations should guide user uptake, implementation, context-specific adaptations and future updates, while preserving the instrument’s core functions across local contexts (i.e., standardizing functions)? Which documentation should accompany the instrument?	Systematic synthesis and triangulation of the validity evidence accumulated across Steps 0–7c. Audit of SC deliberations and metrological decision-making retraced in the decision logbook and meeting minutes. Review of implementation science evidence, guidelines, and frameworks relevant to dissemination, implementation, and scale-up. Dedicated work group for packaging the final instrument (including drafting the test manual, dissemination and implementation plan, training requirements, and adaptation framework, where applicable).	A methodological publication could document the complete IDP development process, including key metrological decisions and the validity evidence accumulated throughout the protocol. The finalized IDP instrument, administration manual, scoring guidance, and supporting materials could be disseminated as Supplementary material or through controlled access, where appropriate, to promote appropriate use by qualified users and minimize inappropriate interpretation or application. Alternative IDP formats (e.g., professional, patient/caregiver, and research versions) could be developed while preserving the instrument’s core functions and intended interpretation.	TC, RP, IS, RoV & CoT: The accumulated validity evidence should be transparently synthesized (e.g., graphically through a validity evidence profile) to make explicit the contextual boundaries within which the instrument’s validity is supported, thereby defining the conditions for appropriate use and preserving the instrument’s integrity across context-specific adaptations.

SC = Steering Committee; TC = Evidence Based on Test Content; RP = Evidence Based on Response Processes; IS = Evidence Based on Internal Structure; RoV = Evidence Based on Relations to Other Variables; CoT = Evidence Based on Consequences of Testing.

The protocol is organized into eight iterative steps preceded by a preliminary phase dedicated to establishing a participatory governance structure, implemented here through a multidisciplinary Steering Committee (SC) that remains active throughout the process and serves as its deliberative body (Figure 1). Tables 2–4 provide an overview of each step by presenting: (i) its objective; (ii) the SC’s deliberative questions and guidance for that stage; (iii) illustrative procedures and evidence sources (“Decision Inputs”) that may support and inform the SC’s deliberations; (iv) the anticipated implications of methodological decisions for the five sources of validity evidence described in the Standards for Educational and Psychological Testing, highlighting potential synergies and trade-offs; and (v) illustrative IDP development scenarios, representing plausible methodological directions emerging from the protocol while remaining conditional on the SC’s deliberations and the evidence accumulated throughout the development process.

Several phases of the protocol will involve human participants (e.g., interviews, questionnaires, pretesting, or observational procedures) and will undergo appropriate ethics review before implementation. As these empirical procedures are, by design, not yet fully specified at the current stage of this open participatory protocol, appropriate ethics review will need to be anticipated at each relevant step as the corresponding study procedures are defined. Depending on these procedures, safeguards will include informed consent, confidentiality and privacy protection, secure management of recorded or identifiable data, and specific consideration of participant vulnerability.

### 3.1 Development phase

#### 3.1.1 Preliminary step: Project governance

The Preliminary Step defines how metrological decisions will be governed before instrument development begins. The SC first develops a partnership charter specifying the project’s governance rules (e.g., stakeholder representation, roles, decision-making procedures, conflict management, and decision traceability; Bartholomew Eldredge et al., 2016; Dupoyet et al., 2020; Majid et al., 2018). Because SC composition may shape the definition of the construct itself, and consequently evidence based on test content, stakeholder representation is discussed explicitly from the outset. Stakeholders may legitimately pursue different priorities according to their experience, roles, and expectations, so the IDP project charter will formalize shared values and commitments while recognizing the legitimacy of divergent viewpoints. To facilitate deliberation, the IDP project will designate a facilitator (here, a social psychologist with expertise in psychometrics and group facilitation, and a member of the SC, supported by another SC member where appropriate). When disagreement persists, the SC is expected to request additional Decision Inputs (e.g., among the methods suggested for the relevant development stages; see Tables 2–4), use structured decision-support tools (e.g., priority–feasibility matrices or decisional balance), postpone the decision pending additional evidence, or explicitly acknowledge that no consensus can currently be reached while documenting the competing positions. Within the IDP project (see Table 2), meeting minutes will document decisions, their rationale, and unresolved disagreements, allowing subsequent stages—or future projects—to revisit previous deliberations, pursue alternative pathways, or reopen decisions in light of new evidence. The SC will include at least researchers specializing in social interactions, clinicians with expertise in psychopathology and social disorders, psychometricians, implementation scientists, and patient and family representatives, with membership evolving across development milestones as additional expertise becomes necessary.

#### 3.1.2 Step 1: Intended use

Step 1, at the core of anticipating and documenting evidence based on consequences of testing, will establish the intended measurement functions (i.e., what the instrument is expected to measure, for what purpose, and under which conditions), target users, contexts of use, and interpretive boundaries of the future instrument. These decisions will guide subsequent construct definition, administration procedures, item generation, validation priorities, and implementation planning, and thereby influence the sources of validity evidence that depend upon them (Table 2). The SC will identify which stakeholder needs should be prioritized, determine whether they are mutually beneficial, merely compatible, or potentially conflicting, and decide which trade-offs have to be made. The protocol will retain only those intended uses for which sufficient validity evidence is expected to be accumulated, while anticipating foreseeable unintended consequences. Intended uses that cannot yet be adequately supported (e.g., diagnosis, prognosis, or medico-legal decision-making) may therefore be explicitly excluded or deferred until dedicated validity evidence becomes available. Within the IDP project, these deliberations will primarily rely on the expertise of the SC, but may, where appropriate, also be informed by environmental scans, reviews of existing instruments, and stakeholder interviews or focus groups (Table 2). These deliberations may thus give rise to the ambition of developing the IDP as a single instrument intended to support multiple intended uses, including routine clinical assessment, intervention planning, crisis prevention, self-management, longitudinal monitoring, and research. Explicit safeguards could also be established, specifying conditions for use, non-use, or deferred use. For example, the SC could decide that the IDP should not be used to justify decisions affecting access to care or support services, or to interpret isolated interactional behaviors outside their relational and contextual context, thereby reducing the risk of overpathologization. Whether a single instrument could ultimately support several functions (or whether context-specific versions would be required) will likewise remain conditional upon subsequent SC deliberations and the validity evidence accumulated throughout development.

#### 3.1.3 Step 2: Construct definition

Step 2 will define the construct, its conceptual boundaries, dimensions, and nomological network. As this stage determines what the future instrument is (and is not) intended to measure, it is central to establishing evidence based on test content while preventing construct contamination and overlap with adjacent constructs (Table 2). The SC will first determine whether interactional disruption should be conceptualized as a disorder-specific or transdiagnostic construct. Our current working hypothesis nevertheless favors a transdiagnostic and etiologically neutral conceptualization. Under this hypothesis, a behavior is considered according to its observable form and its perceived effect on the ongoing interaction, rather than according to the person’s diagnosis or presumed causes such as symptoms, medication, stress, or neurological factors. Similar manifestations may therefore occur across clinical conditions and in individuals without a psychiatric diagnosis. The SC will also determine which phenomena belong to the construct and which should remain outside its scope (e.g., verbal content, etiological or psychological inferences, diagnostic interpretations, or moral judgments about the person, whose exclusion would be justified here on conceptual grounds, beyond the use-related grounds for exclusion potentially identified in Step 1). It will also determine which manifestations should be retained (e.g., reduced, excessive, paralinguistic, atypical, poorly timed, or interactionally disruptive behaviors), whether their meaning remains invariant across targeted populations, cultures, and contexts, and which conceptual organization best represents the construct (e.g., unidimensional, multidimensional, hierarchical, or profile-based). Deliberations will additionally address the trade-offs between comprehensiveness, parsimony, flexibility, and interpretability. Because these decisions will influence subsequent development stages and multiple sources of validity evidence, their metrological implications will also be considered (e.g., retaining the initial term *Disruptive Behavior* could have eventually led to stigmatization and adversely affect evidence based on consequences of testing, whereas an overly specified construct could result in an unnecessarily technical and burdensome instrument, thereby compromising evidence based on response processes). These deliberations may, where appropriate, be informed by scoping or systematic literature reviews, concept mapping, reviews of existing instruments, stakeholder interviews or focus groups, and cross-cultural evidence, depending on the project’s needs, available resources, and the expected added value of these decision inputs (Table 2). Without presupposing the outcome of these deliberations, the IDP may be provisionally defined as focusing on directly observable nonverbal and interaction-regulatory behaviors. Its initial organization may draw on the formal distinction between positive and negative symptoms in schizophrenia, whereby positive manifestations would refer descriptively to excessive, intrusive, poorly timed, or contextually incongruent behaviors, whereas negative manifestations would refer to expected interactional signals that are markedly reduced or absent. This distinction would remain descriptive and would not necessarily imply diagnostic equivalence or a predefined factor structure.

#### 3.1.4 Step 3: Administration specifications

Step 3 will determine how the construct should be operationalized into administration format(s) and contexts. Drawing on the distinction between core functions and forms used in complex intervention research (Hawe et al., 2004; Skivington et al., 2021; Villeval et al., 2019), the protocol will require the SC to recall the metrological functions formalized in Steps 1 and 2 that must be preserved across administration modalities. Indeed, the open protocol abandons the assumption that respondents or practitioners should uniformly adapt to a rigid, standardized administration procedure. Instead, it promotes flexibility through either instrument calibration to the measurement context or the development of functionally equivalent administration forms. Such flexibility is particularly relevant to interactional disruption, whose authentic expression may be altered by overly visible or artificially arranged assessment conditions, thereby compromising the validity of the assessment (Table 2). Within the IDP project, priority may therefore be given to adaptability (e.g., a versatile instrument, an open administration procedure, or equivalent administration forms), whereas the requirement for standardization may initially be deferred to the interpretation of scores (e.g., standard scores, normative scaling, or calibration procedures; see Step 4). Inspired by the administration strategy of the CAINS, the IDP could, for example, combine a semi-structured interaction interview format with standardized behavioral ratings assigned immediately afterwards from behaviors observed during the interaction, thereby avoiding disruption of the interaction while limiting retrospective recall bias. The SC may also determine whether interactional disruption should be assessed from a single interaction or across repeated observations, and whether routine clinical observations are sufficient or should be complemented by more ecological administration procedures (e.g., *video-recorded interactions, interaction tasks, role-plays, diaries, intensive longitudinal observations, or smartphone-based monitoring such as Ecological Momentary Assessment*; Wrzus and Neubauer, 2023). Such decisions may depend on whether the objective is to capture stable interactional tendencies, context-dependent manifestations, or their variability over time. The IDP could therefore also be implemented through a video-recorded interaction procedure for behavioral review or clinical longitudinal follow-up, provided that functional equivalence is empirically demonstrated. Different administration modalities (e.g., paper-and-pencil, clinician-rated, smartphone-based) and inclusive formats (e.g., image-based formats for lower literacy populations) could be considered. Once again, when no administration strategy appears clearly preferable, the SC may draw on the broader range of Decision Input procedures available for this step (Table 2). In particular, collaborative ideation techniques (e.g., structured brainstorming) or co-design workshops may be used to generate alternative administration concepts before selecting the most promising option(s).

#### 3.1.5 Step 4: Candidate items generation

Step 4 will translate the construct established in Steps 2 and 3 into a preliminary pool of candidate indicators, items, response formats, and coding strategies. Information accumulated during the previous steps will be operationalized into an initial pool of candidate items informed or derived from theory and evidence-based data, including empirical findings, experiential knowledge, and cross-mapping of existing tools (Table 2). For example, instruments such as the CAINS, or the Motivational Interviewing Skill Code (MISC), which operationalizes the interaction itself through the coding of reciprocal clinician–patient exchanges rather than isolated behaviors emitted by either interaction partner, may provide valuable sources of inspiration for developing the future IDP. Building on these elements, the SC will then determine how the construct is best represented by deciding which manifestations should be retained, whether they are best operationalized as isolated indicators, behavioral patterns, or reciprocal interactional sequences, and under which contextual conditions they should be interpreted. Because the same behavior may be clinically meaningful, adaptive, or entirely unremarkable depending on its timing, reciprocity, frequency, or interactional context, candidate indicators will be developed to preserve the meaning of the construct as expressed within the administration conditions defined in Step 3. This issue may become particularly salient if the future IDP is hetero-rated, as the same behavior may be perceived as disruptive by clinicians sensitized to subtle warning signs, whereas relatives may progressively normalize or accommodate the same behavior, and therefore no longer perceive it as disruptive. In other words, the operationalization should preserve not only the construct itself, but also its expression and interpretation within the administration context. Particular attention will therefore be devoted to wording, contextual interpretability, usability, response processes, coding principles, and preliminary scoring options.

As a prospective illustration for the IDP project, Supplementary Table S1 provides preliminary candidate domains and illustrative items operationalized as observable behavioral indicators of interactional disruption. These provisional indicators encompass facial expressions, gaze behavior, gestures, posture, interpersonal distance, turn-taking, backchannels, and other interaction-regulatory behaviors while distinguishing descriptive positive manifestations (presence, excess, intrusion, poor timing, or contextual incongruence) from negative manifestations (reduction or absence), without presupposing dimensionality or scoring architecture. Although this preliminary material may itself constitute a Decision Input, it will remain for the SC to deliberate on its relevance, completeness, and suitability for the intended measurement functions (e.g., whether the initial item pool should remain broad enough to include uncertain or overlapping behaviors, or whether it should focus on visual nonverbal behaviors alone or also include prosody and other paralinguistic features). The SC will also deliberate on candidate coding and scoring strategies. Particular attention will be devoted to score interpretation. For example, an identical total score may reflect either numerous mildly disruptive behaviors or a small number of highly disruptive manifestations despite representing very different clinical situations. Alternative scoring logics, including weighted (e.g., quadratic) response formats, contextual annotations and clinically grounded descriptors, may therefore be explored. The SC may also compare alternative coding strategies, for example combining a global interactional disruption rating (self-, informant-, or observer-rated) with a complementary network analysis of the behavioral organization underlying that rating, before selecting the most appropriate metrological solution for subsequent pretesting.

### 3.2 Validation phase

#### 3.2.1 Step 5: Content validation

The fifth stage aims to document validity evidence related to test content by examining whether candidate items adequately represent the construct of interactional disruption across perspectives while identifying priorities for refinement before subsequent pretesting (see Table 3). A two-round TRIAGE consensus procedure (Technique for Research of Information by Animation of a Group of Experts; Gervais and Pépin, 2002) would be implemented with approximately 6–10 external experts selected according to criteria similar to those used for the SC (e.g., clinicians involved in social interaction disorders or rehabilitation, researchers in nonverbal communication or social interaction, patients, relatives, or other stakeholders, depending on the intended applications of the future IDP). Experts would first complete an online questionnaire evaluating item relevance (e.g., essential, useful but not essential, not essential; Lawshe, 1975) and could suggest modifications, contextual clarifications, or additional candidate items. A second collective discussion phase would then focus on newly proposed items and those lacking consensus. Content Validity Ratios (CVR) would be calculated using thresholds adapted to the number of experts consulted (Ayre and Scally, 2014). Within the TRIAGE procedure, these indices would primarily serve as Decision Inputs to reduce the initial pool of candidate items and structure subsequent SC deliberations. External experts would therefore provide consultative input, whereas the SC, having progressively accumulated and integrated the theoretical, empirical, and experiential evidence gathered throughout the previous steps, would remain responsible for the final decisions. Accordingly, although items with the highest CVR would generally be prioritized, the SC could retain, reformulate, or discard any candidate item whenever the triangulation of available evidence justifies doing so. Face validity procedures could also be conducted according to the same principle with patients or relatives to examine whether items appear relevant, exhaustive, representative, and acceptable from their experiential perspective.

Following these deliberations, candidate indicators could ultimately be reformulated, merged, split, contextualized, relocated, retained, or discarded before subsequent pretesting. These decisions would be made case by case by considering not only evidence based on test content, but also their likely downstream consequences for the other sources of validity evidence. For example, clarifying an item to improve response processes may inadvertently alter its conceptual meaning, whereas retaining a clinically important but potentially stigmatizing indicator may require particular attention during subsequent pretesting. Complete consensus is neither realistic nor the objective. For example, clinicians may consider an item important while patients or relatives find it unclear, stigmatizing, or irrelevant. In such situations, the SC may either postpone its decision pending additional Decision Inputs generated during subsequent steps or decide to retain one option while explicitly acknowledging the associated uncertainties and trade-offs. Divergent viewpoints would therefore be documented together with the rationale underlying the SC’s decisions, including any remaining compromises and uncertainties. In the absence of a consensual solution, this documentation would allow future users to understand the remaining unresolved issues, their likely implications and the adaptations or precautions that may be required for appropriate administration and interpretation for their own context of use.

#### 3.2.2 Step 6: Pre-test

Step 6 aims to examine how future users understand, interpret, and implement the instrument in practice. Beyond item clarity, this phase seeks to document validity evidence based on response processes by identifying usability issues, implementation difficulties, and potential unintended consequences. Because response-process evaluation depends on the administration format retained during the previous steps, it would be premature to prescribe a single procedure. Assuming that, as currently anticipated for the IDP, the instrument is developed as a hetero-administered observational tool, cognitive interviews could be conducted with approximately 6–10 intended users (e.g., clinicians and/or relatives, depending on the retained administration format). Participants would first use the provisional IDP while observing standardized video-recorded clinical interactions or excerpts, either verbalizing their reasoning during administration (think-aloud) or participating in a structured debriefing immediately afterwards. The interviewer would explore how ratings were produced, which behavioral and contextual cues were considered, and which misunderstandings, hesitations, ambiguities, or practical difficulties emerged during administration (e.g., whether intended users can understand and apply each item without real-time guidance and whether the IDP is easy to learn and use). Response-process evaluation would also examine whether users describe observable behavior and its possible interactional impact, rather than inferring intention, personality, diagnosis, or etiology from the behavior. For example, and as experienced, some clinicians may under-rate behaviors they consider pharmacologically or diagnostically explained, whereas the intended measurement concerns their observable interactional consequences rather than their presumed etiology. Qualitative analyses would seek to identify recurring patterns of misunderstanding, inconsistent interpretations, usability issues, or administration difficulties requiring revision of the instrument, its administration procedures, coding instructions, response formats, or scoring principles. Approximately ten interviews are initially anticipated, although additional interviews may be conducted until qualitative saturation is achieved (Leung, 2015; Lincoln and Guba, 2011; Miles and Huberman, 1994; Saunders et al., 2018). Finally, each proposed revision would be examined in light of its likely downstream consequences for the other sources of validity evidence before being retained. For example, explicitly instructing users to disregard the presumed causes of a behavior may improve construct fidelity while requiring additional training or reducing ecological usability. Besides, substantial revisions may require an additional round of cognitive interviews before proceeding to psychometric evaluation, or even prompt a reconsideration of the construct definition and its operationalization (returning to Steps 2–4).

#### 3.2.3 Step 7: Psychometric evaluation (pilot, validation and cross-validation)

Step 7 aims to progressively accumulate psychometric evidence supporting refinement, validation, and cross-validation of the future IDP while examining whether the retained measurement/scoring architecture remains consistent with its intended measurement functions. Pilot, validation, and cross-validation studies would be conducted using representative clinical samples (S1–S3), each informing subsequent SC deliberations (Table 3). Sample size will be determined separately for each of the three psychometric samples, based on the analyses planned at that stage, the measurement model retained, and its metrological context, as no universal rule applies (Gunawan et al., 2021). As these features will progressively emerge throughout the protocol, neither the psychometric procedures nor the resulting sample-size requirements can be fully specified *a priori* for the future IDP. In Step 7a (pilot), the most demanding procedures would likely concern reliability estimation and exploratory analyses. Among available sample-size determination methods, estimation- and precision-based approaches would therefore be preferentially used, with simulation-based approaches where model complexity warrants them. In Step 7b (validation), sample size would instead be primarily informed by model-specific power requirements for confirmatory analyses and hypothesis testing involving external variables, whereas Step 7c (cross-validation) would primarily rely on precision requirements for independent replication and, where applicable, score-interpretation analyses. The psychometric procedures themselves would likely include, as core analyses, sensitivity analyses (e.g., Ferguson’s Delta, D-index), reliability analyses (e.g., test–retest reliability, inter-rater reliability, formal equivalence, internal consistency coefficients), analyses of internal structure (e.g., EFA, CFA, Rasch, IRT, or Generalizability Theory, where appropriate), and analyses of relations with external variables. Additional procedures (e.g., responsiveness, measurement invariance, ROC analyses) could be implemented depending on the retained instrument and its intended measurement functions.

The future IDP will likely be developed as a hetero-evaluated and potentially multi-informant instrument. Accordingly, inter-rater reliability procedures could be implemented. For example, three to four raters could independently assess approximately 40 contextualized behavioral video segments using standardized assessment procedures to evaluate inter-rater reliability and compare alternative measurement/scoring architectures (e.g., latent dimensions, profile-based assessment, or behavioral network analysis), depending on whether the intended interpretation is best supported by a global score, domain-specific scores, a contextualized behavioral profile, or several complementary outputs. These ratings could be assisted by synchronized motion-tracking technologies (Vattier et al., 2026), allowing each Critical Participation Time (CPT) to be examined together with its immediate interactional context. Functional equivalence could additionally be examined across clinician-, relative-, or proxy-rated administration formats, whereas measurement invariance procedures could assess whether the retained measurement/scoring architecture functions similarly across patient profiles, clinical situations, or administration contexts. Regarding evidence based on relations to other variables, convergent, discriminant, criterion-related, and/or predictive validity could be examined according to the nomological network established during Step 2. Depending on the retained hypotheses, external variables will likely include the CAINS (Kring et al., 2013), PANSS (Kay et al., 1987), WILL (Coyne, 1976), and/or AIMS (Guy, 1976).

Psychometric findings would constitute additional Decision Inputs for the SC and would be interpreted alongside the validity evidence accumulated throughout the previous steps to refine, qualify, or, where necessary, reconsider earlier metrological decisions. As part of the future IDP project, psychometric indices and decision thresholds (moreover already extensively documented in the psychometric literature despite ongoing debate regarding their interpretation) would not be applied automatically. Rather, they would be contextualized and interpreted through triangulation with conceptual, clinical, practical, experiential, and ethical considerations. For example, an item with a discrimination index below a conventional cut-off may nevertheless be retained if it captures an uncommon but highly characteristic pattern of interactional disruption whose identification substantially improves behavioral characterization, differential assessment, or clinical decision-making despite its low frequency (i.e., Internal Structure versus Test Content and Consequences of Testing). Similarly, a scree plot may support a less parsimonious factor solution, yet this statistically more informative structure may not be retained if the additional factors cannot be meaningfully conceptualized, translated into distinct behavioral dimensions, or support decisions that differ in clinical practice (i.e., Internal Structure versus Test Content and Consequences of Testing). Likewise, multiplying highly similar behavioral items may indeed improve internal consistency while increasing redundancy, administration burden, and reducing usability (i.e., Internal Structure versus Response Processes). Finally, as a further illustration, cross-loading items may, once again, prompt additional SC deliberation and complementary Decision Inputs before any revision or exclusion is retained, illustrating that psychometric evaluation is not an endpoint, but an integral part of the iterative development and validation of the instrument.

### 3.3 Production phase

#### 3.3.1 Step 8: Test production and writing of the test manual (implementation planning)

The final stage concerns production of the instrument, drafting of the test manual, and preparation of implementation recommendations (see Table 4). This phase aims to consolidate the validity evidence accumulated throughout development and translate it into implementation resources supporting the appropriate interpretation and use of the IDP, while explicitly documenting remaining uncertainties, unresolved issues, methodological limitations, and contextual restrictions. The SC will develop administration recommendations, scoring procedures, interpretative guidelines, and recommendations regarding appropriate and inappropriate uses of the IDP, together with safeguards against unsupported interpretations and misuse. The manual may additionally include standardized training materials, contextualized clinical examples, video-based illustrations, supervision recommendations, and practical decision aids to support appropriate administration, interpretation, and implementation of the instrument. Particular attention will also be devoted to documenting the anticipated consequences associated with the intended and unintended uses of the IDP, in accordance with current testing standards. The manual will be conceived as a structured but flexible guide supporting clinical decision-making rather than replacing professional judgment, since no manual can anticipate all clinical situations (Gilmore et al., 2024). Depending on psychometric results, standardized norms, calibration procedures, or Receiver Operating Characteristic (ROC) analyses may also be proposed to assist score interpretation. Finally, implementation strategies and conditions of use would be specified to facilitate appropriate dissemination in research and clinical practice while preserving the validity evidence accumulated throughout development during routine implementation.

## 4 Anticipated outcomes and methodological contributions

The primary anticipated outcome of this protocol is the progressive development of the Interactional Disruption Profile (IDP) through an open participatory process in which its metrological specifications emerge from successive evidence-informed deliberations rather than being exhaustively specified *a priori*. Accordingly, it may legitimately lead to alternative or complementary IDP versions (e.g., modular, multimodal, or context-specific versions), provided that they preserve shared measurement functions across contexts (i.e., test equivalence). More broadly, the protocol proposes an operationalization of the Standards for Educational and Psychological Testing that more closely reflects their underlying epistemological principles. The Standards themselves are not reconsidered but reaffirmed as a call for greater openness to the relative, contextual, and evolving nature of validity. In line with this perspective, the proposed open protocol departs from the widespread tendency in both psychometric instrument development and intervention research to manage uncertainty through risk-averse, standardized, and prescriptive development protocols, in which key decisions are specified before the project begins. Such standardization may prematurely constrain reflexive deliberation, thereby reproducing the same blind spots and limitations that participatory development seeks to overcome.

A corollary of such openness is that participatory development will inevitably generate situations that cannot reasonably be anticipated before stakeholder involvement. Some questions may have no single optimal solution, requiring explicit compromises between competing sources of validity evidence, whereas others may reveal persistent disagreements between stakeholders, disciplinary perspectives, institutional constraints, or policy priorities (see Preliminary Step). Rather than attempting to illusorily anticipate every possible situation through standardized protocols, the proposed approach is expected to promote adaptability and context-sensitive decision-making within a structured framework that makes uncertainty explicit and enables its deliberation and traceability, thereby enhancing the trustworthiness of the development process. This perspective would be consistent with contemporary approaches to the planning of complex health interventions.

## 5 Discussion

This article aimed to propose an open participatory protocol for the ecological development and validation of the Interactional Disruption Profile (IDP), while examining how current psychological testing standards may be applied to the assessment of context-dependent interactional phenomena. The protocol provides a structured pathway for progressively defining the IDP’s intended functions, construct boundaries, administration procedures, scoring architecture, validity evidence, and conditions of implementation. Its main contribution lies in maintaining these components open to evidence-informed deliberation without leaving their development methodologically unspecified.

A central challenge in developing participatory protocols is to reconcile methodological rigor with openness. A protocol must provide sufficient structure to make the planned development process scientifically assessable, without predetermining methodological choices that should emerge from stakeholder deliberation and accumulating evidence. Rather than prescribing these choices in advance, the proposed open protocol for IDP provides a semi-structured decision-making framework for managing uncertainty. At each stage of IDP development (i.e., *When*), it identifies the fundamental questions to be addressed (i.e., *What*), points to an illustrative repertoire of methodological procedures and evidence sources to inform deliberation (i.e., *How*), and highlights the expected implications of alternative methodological options for the different sources of validity evidence (i.e., *With what implications*). The SC will remain responsible for determining, on a case-by-case basis, which stakeholders (i.e., *Who*) are best suited to address or even take the lead on each question as it arises. This approach is consistent with the Standards for Educational and Psychological Testing, which conceptualize validity as an evolving argument concerning specific interpretations and uses rather than as a fixed property acquired once and for all by an instrument (American Educational Research Association et al., 2014).

The protocol also broadens the role assigned to stakeholder participation in instrument development. Stakeholders are not involved only to confirm predefined content or establish consensus after the main decisions have been made. Through the Steering Committee, their deliberations may challenge initial assumptions, reveal contextual or implementation constraints, and generate metrological solutions that would not necessarily emerge from conventional development routines. This function is consistent with participatory approaches that treat stakeholder knowledge as a component of research design and interpretation rather than as a late-stage consultation exercise (Duea et al., 2022; Vaughn and Jacquez, 2020). Documenting disagreements, rejected alternatives, and successive trade-offs additionally produces knowledge about the developmental history and intended functions of the IDP, which may later support its interpretation, deployment, and context-sensitive adaptation. Such documentation also contributes to the trustworthiness of the development process by making the evidential and deliberative basis of decisions open to appraisal (Lincoln and Guba, 2011).

A further contribution lies in making the relationships among the five sources of validity evidence visible throughout development. As shown, decisions intended to strengthen one source of validity evidence may alter the conditions supporting others (e.g., improving Internal Structure through redundant items may simultaneously increase respondent burden and weaken Response Processes). The widespread use of rigidly standardized administration procedures illustrates this tension well. Such procedures have traditionally been used to safeguard the psychometric qualities of an instrument, particularly evidence based on Internal Structure and Relations to Other Variables, by keeping administration procedures unchanged. However, maintaining such rigid standardization may itself come at the expense of other sources of validity evidence. Thus, requiring respondents or clinicians to adapt to fixed administration procedures that do not correspond to their situation or usual functioning may constrain natural response processes, reduce the ecological expression of the phenomenon under observation, and ultimately weaken evidence based on Test Content, Response Processes, and Consequences of Testing. These tensions are particularly salient when the target construct cannot be separated from the context in which it is expressed (Truijens et al., 2019; Uher, 2022), as is the case for interactional disruption. One possible way forward would therefore be to calibrate administration procedures to the respondent and the measurement context rather than requiring respondents to adapt to the instrument. In this perspective, it would no longer be the administration procedure that guarantees the interpretability and comparability of scores, but the metrological work performed after administration. Standardization would therefore not be abandoned but displaced: from administration forms to equivalent metrological functions, and subsequently toward efforts to reconstruct the interpretability and comparability of scores (e.g., through standardized scores, calibration, normalization, or equating procedures). The open participatory protocol proposed here for the development of the IDP therefore does not reduce validity to a binary distinction between valid and invalid instruments. Instead, it supports an explicit appraisal of the resulting validity profile and of the contextual boundaries within which particular interpretations and uses may be justified, in line with the use-oriented validity perspective endorsed by current testing standards (American Educational Research Association et al., 2014).

This approach also clarifies the role of protocols when uncertainty cannot reasonably be removed before a project begins. The ecological development of the IDP requires both an explicit framework and the capacity to adapt decisions to emerging evidence, stakeholder perspectives, and implementation conditions. Similar reasoning underpins contemporary approaches to complex health interventions, in which adaptation is not necessarily considered a deviation from rigor but may constitute a necessary response to interactions between intervention components, populations, settings, and implementation conditions (Skivington et al., 2021). The protocol therefore prepares the project for context-sensitive decision-making rather than attempting to replace such decisions with exhaustive anticipation. More broadly, the protocol prepares the project not only for context-sensitive decision-making but also for context-sensitive measurement, in which flexibility concerns administration procedures whereas standardization is progressively reconstructed through metrological equivalence and score interpretation.

Several methodological implications and limitations follow directly from this openness.

First, the protocol cannot guarantee that the resulting instrument will ultimately prove valid, feasible, or clinically useful. These properties must be established empirically within each development project. Likewise, the protocol cannot guarantee consensus or a single optimal solution. Persistent disagreements, competing validity arguments, and institutional or practical constraints may require explicit compromises or leave some questions provisionally unresolved. Certain methodological issues may also remain only partially resolvable, particularly those related to contextual variability, cultural interpretation, and construct instability. This makes the quality of facilitation, stakeholder representation, and decision documentation central to the trustworthiness of the process. Second, the protocol remains resource-intensive and requires sustained stakeholder involvement (although these requirements will be partially supported within the IDP project through ANR funding; Grant No. ANR-22-CE17-0036). Iterative deliberation and the collection of additional decision inputs may require more time, expertise, and resources than a fixed development pathway. However, these additional demands also create the conditions for detecting metrological blind spots and avoiding premature closure around familiar solutions. Third, some context-dependent manifestations may remain difficult to operationalize reliably without reducing the ecological complexity that gives them meaning, reflecting a broader tension between standardization and contextual sensitivity in cross-context assessment (Van de Vijver, 2016). Finally, different projects (or successive phases of the IDP project) may legitimately adopt different procedures. The transferability of the protocol therefore rests less on reproducing identical decisions than on preserving the explicitness of the guiding principles, the evidence considered, and the rationale underlying adaptations. This conception is also consistent with the view that test manuals and standardized procedures should support, rather than replace, informed professional judgment in context (Gilmore et al., 2024).

## 6 Conclusion

Developing ecologically grounded assessment instruments for observable nonverbal behaviors that may contribute to interactional disruption raises challenges extending beyond conventional psychometric optimization. The IDP open protocol proposes a semi-structured and iterative roadmap intended to address these challenges progressively by integrating ecological validity, stakeholder participation, response processes, contextual interpretation, and psychometric evaluation within a unified development procedure. Rather than treating ecological concerns as secondary implementation issues, the protocol assumes that they constitute foundational components of psychometric development itself. Within this perspective, methodological uncertainty becomes not a limitation to eliminate prematurely, but a central dimension to document, question, and integrate throughout the construction of clinically meaningful assessment instruments.

## Data Availability

The original contributions presented in the study are included in the article/Supplementary material, further inquiries can be directed to the corresponding author.
