# Supplementary material for: An open participatory protocol for the ecological development and validation of the interactional disruption profile
Source: Front Psychol. 2026 Oct 5;17:1889253. doi: 10.3389/fpsyg.2026.1889253 (PMC13677937; doi:10.3389/fpsyg.2026.1889253)
Supplement: Supplementary file 1 [file Table_1.DOCX]

**Supplementary Table S1**

**Preliminary Candidate Domains and Behavioral Examples for the Interactional Disruption Profile (IDP)**

| **Initial domain** | **Candidate item** | **Positive manifestations (presence / excess)** | **Negative manifestations (reduction / absence)** |
| --- | --- | --- | --- |
| **Cues in the face** | **Facial expressions** | Inappropriate facial expression or emotion for the context of the discussion. | Still face: sudden stop of movements and no facial expression (“poker face”). |
|  | **Facial muscle movement** | Grimace; exaggerated facial muscle tension; inappropriate mouth activity. | Reduced facial muscle movement suggesting lethargy or sadness. |
|  | **Gaze behavior** | “Pathological stare”: sustained eye contact becoming uncomfortable; excessive blinking. | Looks away or around the environment and actively avoids social contact. |
|  | **Smiling** | “Fake smile”: the mouth curves upward but the eyes do not show the same emotion. | Never smiles even in a favorable context. |
| **Cues in the body** | **Nodding** | Excessive, frenzied, or contextually inappropriate nodding (e.g., agreement when the context does not call for it). | Rarely or never nods when nodding would be appropriate for agreement or acknowledgement. |
|  | **Head shaking** | Excessive or inappropriate head shaking, including negation in neutral or agreeable contexts. | Rarely or never shakes the head when disagreement or negation would be appropriate. |
|  | **Gesturing** | Overly wide, rough, and rapid gestures. | Crossed arms maintained for a long period; arms alongside the body; absence of gestures accompanying speech; freezing. |
|  | **Fidgeting / agitation** | Arm tremors or agitation, foot tapping, and restlessness transmitting tension. | Rigidity conveying palpable tension. |
|  | **Self-adaptors** | Hand-to-head or hand-to-body movements; clothing adjustment; finger or wrist movements; fumbling; scratching. | Not yet specified. |
|  | **Body position / orientation / posture** | Inappropriate head orientation; torso turning away from the interlocutor; crouching. | Completely withdrawn, slumped in the chair, leaning against a wall, with relaxed muscles. |
|  | **Touch** | Object adaptors: hands in contact with an object, including grabbing and manipulating it. | Not yet specified. |
| **Interaction behaviors** | **Interpersonal distance** | Too close or proxemic invasion: remains very close to the interlocutor and creates discomfort. | Too far, the distance is awkward. |
|  | **Mimicry / contagion** | Not yet specified. | Never expresses the same facial expression as the partner; appears desynchronized. |
|  | **Turn taking** | Cuts off the interlocutor and speaks during the interlocutor’s turn. | Never takes a turn, or delays before taking it, so that the interlocutor must prompt the exchange. |
|  | **Backchannels** | Backchannels that are excessive in frequency or intensity, including when no feedback is required. | Fails to produce backchannels acknowledging the interlocutor’s speech, creating an impression of disengagement. |

*Note. The broad cue domains were initially informed by the pragmatic nonverbal communication framework of Blanch-Hartigan et al. (2018) and adapted to the clinical observations that motivated the IDP project. “Not yet specified” indicates that no candidate example had been formulated at this preliminary stage.*

This table reproduces the preliminary working classification used as the starting material for the future IDP. Positive manifestations refer descriptively to behaviors characterized by presence, excess, intrusion, poor timing, or contextual incongruence; negative manifestations refer to expected behaviors that are reduced or absent. The original overlap between items, unequal levels of specificity, uncertain wording, and incomplete entries are intentionally retained because the table is not a finalized item pool or an established theoretical structure.
